# Treatment of Recurrent Undifferentiated Pleomorphic Sarcoma of Infratemporal Fossa by Surgery Combined With Carbon Ion Radiotherapy: One Case Report

**DOI:** 10.3389/fsurg.2021.693774

**Published:** 2021-08-10

**Authors:** Zaixing Wang, Zhiyuan Tang, Hailiang Zhao, Xianhai Zeng, Xiaodong Han, Qiuhang Zhang

**Affiliations:** ^1^Institute of ENT and Shenzhen Key Laboratory of ENT, Longgang ENT Hospital, Shenzhen, China; ^2^Center of Skull Base Surgery, China INN and Department of Otolaryngology-Head and Neck Surgery, Xuanwu Hospital, Capital Medical University, Beijing, China

**Keywords:** undifferentiated pleomorphic sarcoma, infratemporal fossa, operation combined with carbon-ion radiotherapy, otolaryngology head and neck surgery, recurrent

## Abstract

We retrospectively analyzed the diagnosis and treatment process of one patient with recurrent undifferentiated pleomorphic sarcoma (UPS) of infratemporal fossa and made a definite diagnosis by combining the imaging and pathological examination results. After treatment failure with 2 cycles of chemotherapy and several surgeries, UPS was eventually treated by surgery + carbon ion radiotherapy, and MRI reexamination showed no relapse. Head and neck UPS is located deeply, easily recurs after operation, and difficult to be resected completely by surgery, with a gradually shortened interval of relapse over the number of surgeries, which becomes a treatment challenge. After the last surgery, the patient received carbon ion radiotherapy, with a good therapeutic effect, and no sign of relapse just before sending this article. Based on the above advantages, we have concluded that surgery + carbon ion radiotherapy is a new effective pathway to treat head and neck UPS.

## Highlights

- Surgical excision of UPS alone cannot achieve the goal of cure.
- The UPS of head and neck region is not sensitive to chemotherapy.
- The UPS could be controlled and cured by operation with carbon-ion radiotherapy, However, long-term efficacy remains to be seen.

## Introduction

Undifferentiated pleomorphic sarcoma (UPS) may occur in any organ, most frequently in soft tissues of limbs and retroperitoneal space, but rarely in head and neck (higher malignancy, easier relapse and metastasis, and poorer prognosis); it originates from mesenchymal tissues, and its incidence rate accounts for 1% of all malignant tumors (1). One case of recurrent UPS treated by surgery + carbon ion radiotherapy was reported to explore the clinical significance of such new combination.

## Case Report

The patient, female, 60 years old, was admitted to the hospital due to nearly 2 months after several surgeries for UPS of left infratemporal fossa and just after 1 cycle of chemotherapy. In January 2016, the patient felt sore and bursting at left cheek but paid no attention and received no examination and treatment.

**Figure 1:**
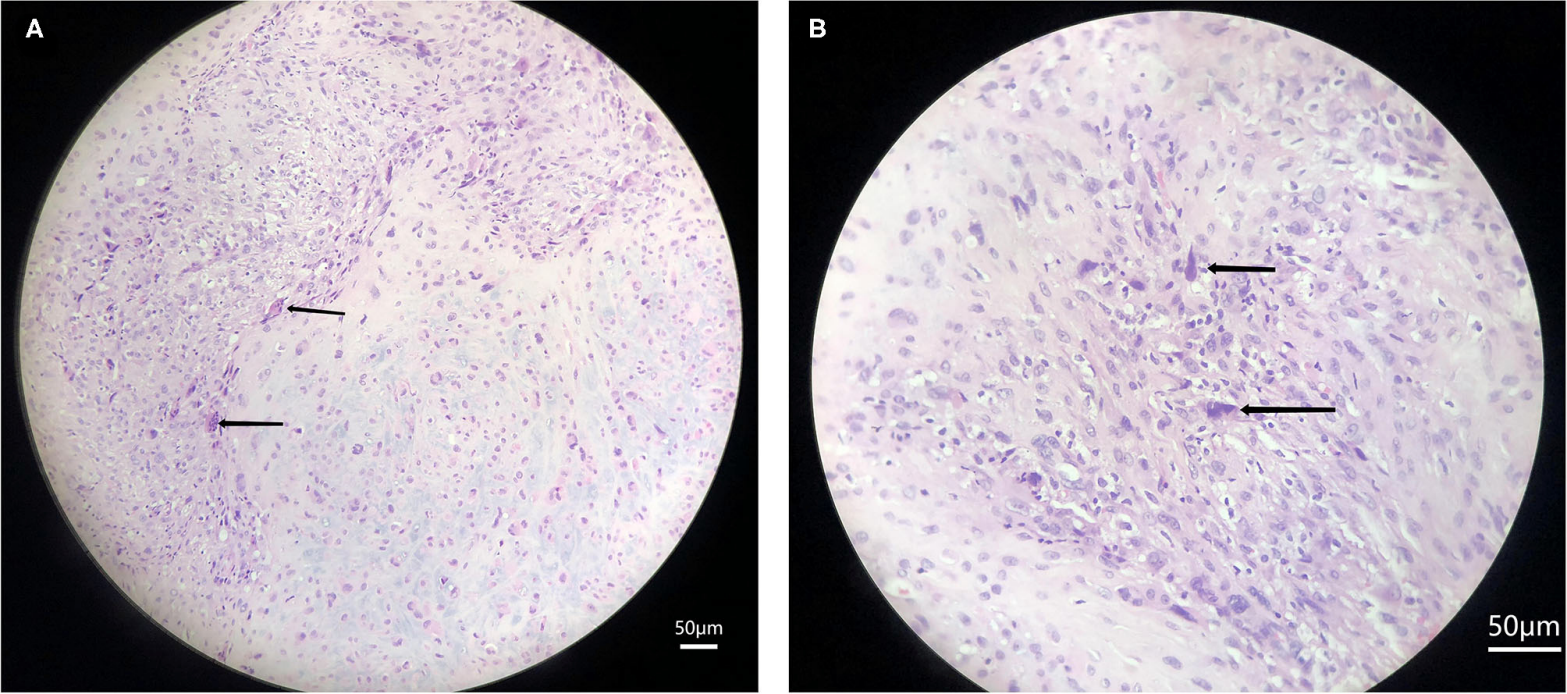
Pathological slice of undifferentiated pleomorphic sarcoma. Arrows indicate that the tumor cells have obvious nuclear atypia and pathological nuclear mitosis. **(A)** 20X HE stain, **(B)** 40X HE stain.

On 7 March 2016, the patient paid a medical visit to Otolaryngology Hospital—the First Affiliated Hospital, Sun Yat-sen University, and then received rhinoscopic skull base tumor biopsy. The pathological examination results of HE-stained tissue sections indicated that tumor cells were short fusiform or elliptical and significantly atypical, with visible pathological mitosis, mononuclear or multinuclear tumor giant cells, thus morphologically complying with malignant tumor; immunohistochemistry (tumor cells) showed Vimentin (+), desmin (less) (+), Ki67 50% (+), CK (–), GFAP (–), S-100 (–), CD34 (–), Actin (–). The above findings met the diagnostic criteria of (skull base) UPS (Figures 1A,B).

**Figure 2:**
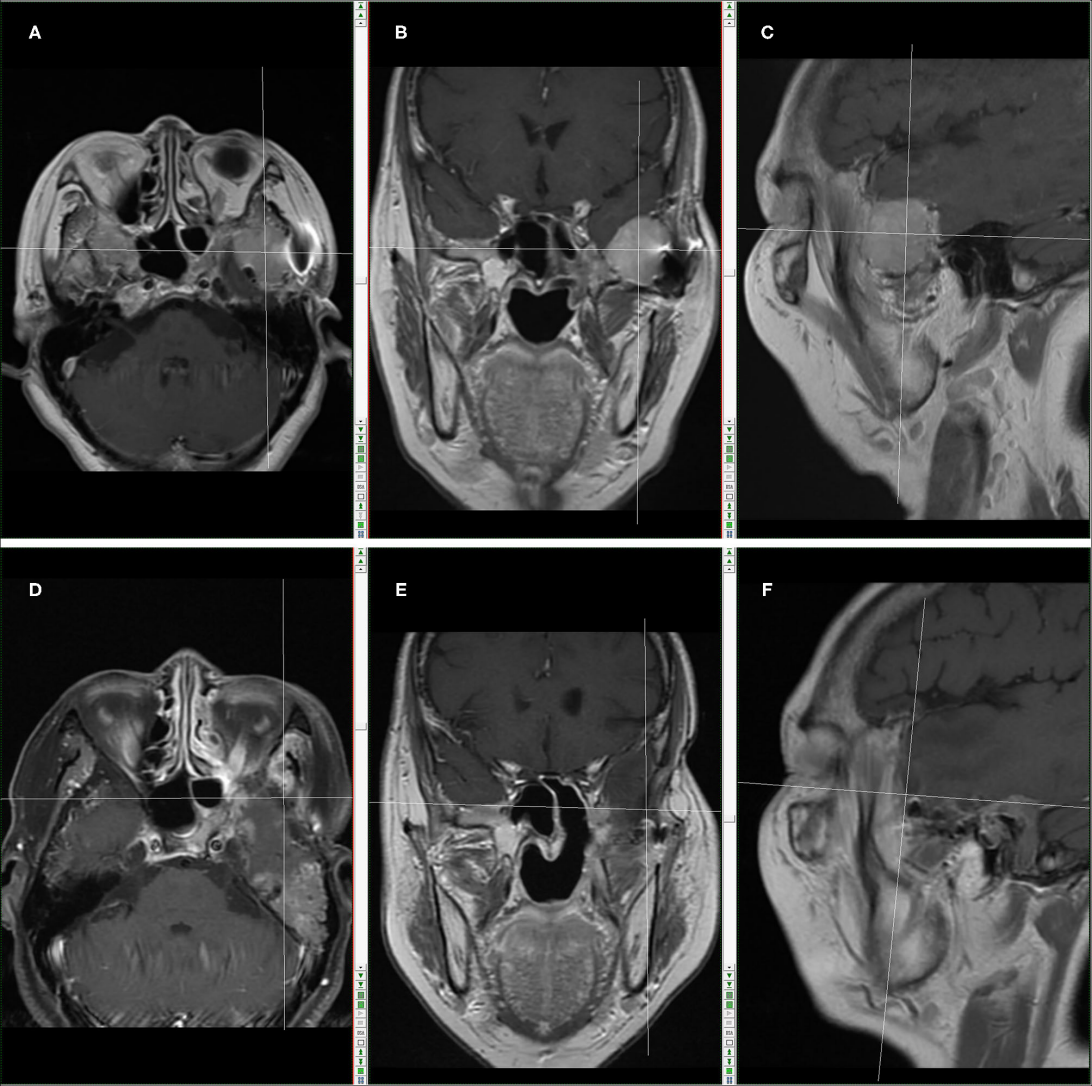
Skull base MRI in May, 2018 (Before the operation combined with carbon-ion radiotherapy): **(A)** MR images in transection. **(B)** MR images in coronal plane; **(C)** MR images in sagittal plane. The cross location denotes the recurring tumor. Skull base MRI in March, 2019 (After the operation combined with carbon-ion radiotherapy): **(D)** MR images in transection; **(E)** MR images in coronal plane; **(F)** MR images in sagittal plane. The cross location denotes no recurrent tumor.

On 5 and 25 April 2016, the patient successfully completed 2 cycles of chemotherapy with IFO+mesna+doxil in Sun Yat-sen University Cancer Center. Skull base MRI examination in May 2016 showed compression-caused displacement of left temporal lobe, and a mass shadow in left infratemporal fossa—middle cranial fossa, with non-uniform signals, predominantly T1 and T2 equisignals in the lesion, patchy slightly low T1 and significantly high T2 signals in the lesion center, nearly a clear and smooth border, and localized mild lobulation, in a size of 30.5 mm (AP) × 39.2 mm (LR) × 29.5 mm (HL), and surrounded by multiple circuitously routing flowing-void vascular shadows. Enhanced scan indicated a significantly enhanced mass in left infratemporal fossa—middle cranial fossa, a patchy low signal non-enhancement area in the center, and linear enhancement nearby meninges. The size of lesion had no significant change as compared with before chemotherapy (Figure 2).

On 25 May 2016, the patient underwent endoscopic transnasal resection for left infratemporal fossa—middle cranial fossa tumor in Otolaryngology Hospital (Longgang District, Shenzhen, Guangdong, China), and the postoperative pathology indicated that the lesion complied with UPS. After operation, the lesion recurred.

On 13 October 2016, the patient underwent subtemporal-preauricular resection for left infratemporal fossa—middle cranial fossa tumor in the same hospital, and the postoperative pathology indicated that the lesion complied with common osteosarcoma (co-presence of chondroblastic osteosarcoma and fibroblastic osteosarcoma). After operation, the lesion recurred.

On 14 October 2017, the patient underwent subtemporal-preauricular resection for left infratemporal fossa—middle cranial fossa tumor in the same hospital, and the postoperative pathology indicated that the lesion complied with common osteosarcoma. After operation, the lesion recurred again.

According to skull base MRI examination in May 2018, there were changes after surgeries for left infratemporal fossa—middle cranial fossa tumor, local bone defect in left maxillary sinus wall, cribriform plate, sphenoid bone and middle nasal concha, compression-caused displacement of left temporal lobe, and an enlarged mass shadow in left infratemporal fossa—middle cranial fossa, with non-uniform signals, predominantly T1 and T2 equisignals in the lesion, patchy slightly low T1 and significantly high T2 signals in the lesion center, nearly a clear and smooth border, and localized mild lobulation, in a size of 35.6 mm (AP) × 42.3 mm (LR) × 35.9 mm (HL), and surrounded by multiple circuitously routing flowing-void vascular shadows. Enhanced scan indicated a significantly enhanced mass in left infratemporal fossa—middle cranial fossa, a patchy low signal non-enhancement area in the center, linear enhancement nearby meninges, a few long T2 abnormal signals in left middle ear and mastoid process, thickened mucosa in bilateral maxillary sinus and ethmoid sinus as well as left sphenoid sinus, a cerebral spinal fluid (CSF)-like signal shadow in sellar region, and compression-caused flattened pituitary. Tumor relapse was considered (Figures 2A–C).

On 21 May 2018, the patient underwent subtemporal (preauricular) resection for left infratemporal fossa—middle cranial fossa tumor in the same hospital. The surgical procedures were as follows: The patient was in a supine position. After successful tracheal intubation and general anesthesia, the patient was routinely kept in a required position with left ear upward, followed by routine disinfection with iodophor and draping. A “C” surgical incision was made at the original incision scar on the hairline of anterior left ear edge and extended to left external acoustic meatus orifice, the skin, subcutaneous tissue and superficial fascia were dissected layer by layer using low-temperature plasma radiofrequency ablation, the skin flap was dissociated, zygomatic arch was exposed at lateral inferior parotid gland, and then its superficial muscular fasciae was dissected. Subsequently, the incision was dilated with a distracter, and the operation space was exposed under endoscope, observing a white fish-like mass with a smooth surface. Then, the mass border was dissected using a stripper upward to cerebral dura mater, and the mass was cut open at the center and mostly resected with a curette. Thereafter, under endoscopic view, the inferior tumor was resected to mandibular ramus and lateral pterygoid of infratemporal fossa and medially to os petrosum, internal carotid artery of os petrosum was exposed, and tumor tissues were fully curetted with a curette at middle cranial fossa base in an up-to-down manner along middle skull base meninges, without residual tumor. During operation, cerebral dura mater of middle cranial fossa base wall was thin, and there were no CSF leakage but slight blood exudation; full hemostasis was performed using bipolar coagulation scalpel, the operation space was rinsed with iodophor and physiological saline and then filled with iodoform gauzes after cerebral dura mater was covered with SURGICEL Absorbable Hemostat and Biodesign Surgisis Dural Graft, these gauzes were withdrawn from external auditory meatus, and the subcutaneous tissue and skin were sutured layer by layer after finishing the surgical count of instruments and gauzes. The surgery was successful, the intraoperative bleeding volume was 1,200 ml, and the resected tumor was sent for pathological examination.

From 16 July to 13 August 2018 after operation, the patient received carbon ion radiotherapy (70 GyE, 20 Fx) for recurrent tumor and treatment-extending safe area. In March 2019, brain MRI reexamination showed no recurrent tumor lesion (Figures 2D–F).

The above examination and treatment items have obtained the informed consent from the patient who participated in clinical investigations.

## Discussion

UPS, also known as malignant fibrous histiocytoma (MFH), is an extremely rare soft tissue malignant tumor. According to WHO latest classification standard in 2013, the concept of MFH was replaced by UPS (2). UPS highly occurs at an age of 60–70 years, with a similar morbidity in males and females; it is frequently seen in limbs, trunk, head and neck, and other organs and tissues, and located deeply, has a high tumor grade and high malignancy, and easily recurs after operation (3). After diagnosis, this case was treated by chemotherapy (no good response) and several surgeries, but the treatment effect was poor, and the patient experienced several relapses with a gradually shortened interval, which complied with the easy recurrence character of UPS.

UPS patients often have a low 5 year survival rate, i.e., 30–50% (3). Peiper et al. (4) investigated 97 patients with a diagnosis of UPS, and their study results showed that the local relapse rate of UPS and the relapse rate of distant metastasis in 13 months (on average) were 31 and 30%, the mean survival was 84 months, and the 5 year survival rate was 70%. Canter et al. (5) also agreed to a low 5 year survival rate of UPS patients as abovementioned. As shown by the study of Lehnhardt et al. (6) which involved 140 cases of UPS in limbs, the 5 year overall survival rate was 72%. In this case, the patient survival could be continuously followed up.

According to the study of Clark et al. (7), head and neck UPS had strong invasiveness and poor prognosis. Sabesan et al. (8) retrospectively analyzed UPS in head and neck and other body parts and drew a conclusion consistent with Clark, which may be attributed to a smaller distance between head and neck tumors and critical structures and a bigger difficulty in surgically resecting complete tumors. Compared with tumors in body trunk and limbs, head and neck tumors are more easily detected at the early stage and thus often have a smaller size and a lower grade. In this case, however, the tumor was located deeply at infratemporal fossa and thus difficult to be completely resected by surgery; considering no distant metastasis, the treatment was surgery + radiotherapy, without requiring chemotherapy.

In the recent years, heavy ion radiotherapy has gradually become a leading technique in the field of tumor radiotherapy, and heavy ions refer to ionized particles heavier than helium element. Carbon ion radiation kills tumor cells via the abovementioned cluster injury, while traditional low-LET-radiation X ray injures tumor cells mainly by independently destroying DNA single-strand or double-strand; tumor cells can repair the injury caused by the later, but are incapable to repair more complicated and diverse DNA cluster injury caused by the former; thus, DNA cluster injury is an important mechanism of carbon ions killing tumor cells (9). In the past nearly 20 years, the National Institute of Radiation Medicine (Japan) has intermittently conducted the clinical studies of carbon ion radiotherapy for head and neck tumors, involving 175 cases of adenoid cystic carcinoma, 102 cases of malignant mucosal melanoma, and 50 cases of adenocarcinoma, separately located in paranasal sinus, nasal cavity, major salivary glands and throat, of which nearly 74% could not be treated by surgery; the 5 year local control rate (LCR) and 5 year overall survival rate (OSR) were 74, 79, 81, and 72, 33, 57%, respectively (10). In another study totally including 76 cases of malignant skull base tumor (44 cases of chordoma, 14 cases of chondrosarcoma, 9 cases of olfactory neuroblastoma, 7 cases of malignant meningioma, 1 case of giant cell tumor, and 1 case of neuroendocrine carcinoma), the treatment was NIRS + carbon ion radiotherapy, the 5-year LCRA and OSR were 88% and 82%, and none of patients experienced serious adverse reactions (11).

The major therapy for malignant tumors of head and neck and skull base is traditionally surgical resection, but complete resection is difficult for tumors with deep invasion and special locations, and surgical treatment will occasionally damage the face of patients and thus seriously influence the quality of life; therefore, radiotherapy is required to improve the LCR. A part of tumors, such as UPS, chordoma and adenoid cystic carcinoma, are resistant to conventional X-rays, and their local control needs a dose of 60 GyE or above (12). In this case, several surgical resections + postoperative 70 GyE radiotherapy realized the purpose of controlling tumor relapse; no sign of tumor relapse was observed before sending this article, and the patient could be continuously followed up. Traditional radiotherapy is incapable to safely release the above dose because of influence from surrounding dangerous organs (spinal cord, brain stem, optic neuropathway, etc.), while carbon ion beam can achieve the local control efficacy, without injuring these organs and changing the face of patients.

In this case, multiple surgical resections and a gradually shortened interval of tumor relapse brought to a challenge for treating UPS, but it was noted that heavy ion radiotherapy after the last surgery achieved a good therapeutic effect, and no sign of relapse was found before sending this article. Based on the above advantages, we have concluded that surgery + carbon ion radiotherapy is a new effective pathway to treat head and neck UPS.

## Data Availability Statement

The raw data supporting the conclusions of this article will be made available by the authors, without undue reservation.

## Ethics Statement

Written informed consent was obtained from the individual(s) for the publication of any potentially identifiable images or data included in this article.

## Author Contributions

ZW, ZT, and QZ: concept and design. ZW, ZT, HZ, XZ, and QZ: supervision. ZW, ZT, HZ, XZ, XH, and QZ: resources. ZW, ZT, XH, and QZ: materials. ZW and ZT: data collection and processing, analysis and interpretation, literature search, and writing manuscript. QZ: critical review. All authors contributed to the article and approved the submitted version.

## Conflict of Interest

The authors declare that the research was conducted in the absence of any commercial or financial relationships that could be construed as a potential conflict of interest.

## Publisher's Note

All claims expressed in this article are solely those of the authors and do not necessarily represent those of their affiliated organizations, or those of the publisher, the editors and the reviewers. Any product that may be evaluated in this article, or claim that may be made by its manufacturer, is not guaranteed or endorsed by the publisher.
